# The Effects of 910-MHz Electromagnetic Field on Rat Cranial Arachnoid and Dura Mater Collagen. The Axial Periodicity of Collagen Fibrils

**DOI:** 10.1100/tsw.2004.180

**Published:** 2004-10-20

**Authors:** Margaret Tzaphlidou, Evangelos Fotiou

**Affiliations:** Laboratory of Medical Physics, Medical School, Ioannina University, 45110 Ioannina, Greece

**Keywords:** electromagnetic radiation, electron microscopy, electron-optical data, image analysis, collagen

## Abstract

The axial periodicity of rat arachnoid and dura mater collagen fibrils exposed to 910 MHz for 2 h/day for 30 consecutive days was measured by means of image analysis of electron-optical data. Such measurements were compared with those from sham-exposed animals. These measurements reveal that on exposure, the intermolecular interactions during collagen fibril assembly are affected.

### INTRODUCTION

It has been established that 900-MHz electromagnetic fields (EMF) induce various measurable biological effects[1] ranging from cutaneous problems[2] to changes in oxidant[3,4] and antioxidant[4] levels, liver[5], epidermal cells[6], brain[7] and DNA[8,9]. EMF influence the synthesis of proteins proportionally to their intensity and duration[10]. Among them, the synthesis of collagen is subject to this influence to a great extent[11].

When collagen fibrils are seen in the electron microscope, they exhibit a characteristic banded appearance (Fig. 1, top). The banding repeats regularly in the direction of the fibril axis with a periodicity D. This periodicity arises because the molecules are assembled in roughly parallel array and are mutually displaced axially by D or integral multiples of D[12]. These axial relationships between molecules in a fibril are shown schematically in Fig. 1, bottom. The scheme is merely a diagrammatic representation in two dimensions and cannot show all possible stagger relationships in three dimensions; in essence, it shows the predicted positions of molecules projected on to the fibril axis.

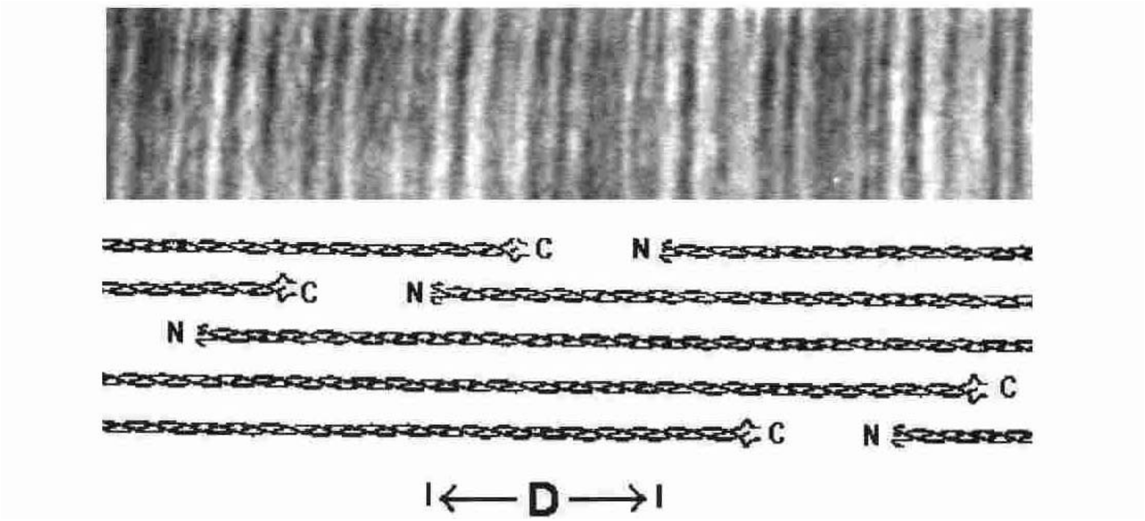

**FIGURE 1.** (Top) A positive staining pattern from arachnoid and dura mater rat collagen fibrils suitably aligned with the corresponding array of D-staggered molecules (bottom). The schematic representation of the axial relationships between molecules in a collagen fibril is based on "Hodge-Petruska" packing arrangement[13].

In this work, we present results for D values from normal and 910-MHz EMF-exposed collagen fibrils (electron-optical images) of arachnoid and dura mater rat brain, as measured by a computer-aided procedure. These measurements aim to seek information to whether or not this frequency influences the intermolecular interactions during collagen fibril assembly.

### MATERIALS AND METHODS

#### Animals

Male Wistar rats were housed in groups of four and were allowed to freely take solid diet and tap water. The breeding room was light (light period from 7:00 a.m. to 7:00 p.m.) and temperature ($20 \pm 1^oC$) controlled. The experiments were started when the rats were either 5 or 16 months of age. For all experiments, 32 animals were used; 16 were 5 months old and the rest 16 months of age. Half of each age group were exposed to radiation and the rest were sham exposed and served as controls.

#### Irradiation Setup

Sixteen animals were exposed for 2 h/day for 30 consecutive days to radiation at 910 MHz. The irradiation setup used consisted of a CW electromagnetic generator with a maximum power output of 2.2 W and a dipole $\lambda/2$ antenna. Each time, four rats were exposed placed per two in two plexiglas cages. Rat cages were placed 5 mm away from the transmitting antenna from both sides in order to ensure a near field and equal exposure of the animals. Dositometric analyses were performed through SAR (specific absorption rates). SAR was calculated by the Finite Difference Time Domain (FDTD) analysis and the maximum value was found to be 0.42 W/Kg averaged over 10 g of tissue. More details for the irradiation setup are given in Tzaphlidou et al.[14]. Rats in groups of four were sacrificed 1 day and 2.5 months after the end of the experiment.

Throughout the experiments, care was taken to minimize pain or discomfort. All studies were approved by the Ioannina University Institutional Animal Care and Use Committee.

### Tissue Preparation

Rats were deeply anesthetized with ethyl ether. The head was cut with a big pair of scissors and the skull was opened with cutting forceps. Brain was removed with fine curved forceps and placed on a Petri dish on ice. Arachnoid and dura mater were gently teased from the surface of the brain and the internal surface of the skull. The isolated menigiel material was placed in Eppendorf tubes containing 2.5% glutaraldehyde buffered to a pH of 7.4 with 0.1 *M* sodium cacodylate. From the material studied, pieces of 1 $mm^2$ were obtained and immersed into the same fixative overnight at 4°C. After this time, samples were washed in 0.1 *M* sodium cacodylate buffer and postosmicated for 2 h at 4°C in 1% osmium tetroxide buffered in cacodylate, followed by washing in the same buffer and finally rinsed in distilled water. The specimens were then dehydrated in a graded alcohol series and impregnated overnight with a 1/1 mixture of propylene oxide and the resin used for embedding. Final embedding was performed in capsules with Agar resin. Polymerization was complete in 48 h at 60°C.

Ultrathin sections were positively stained with 2% aqueous solution of phosphotungstic acid (PTA), pH 3.3 (pH was adjusted with 0.1 *N* NaOH), for 1 h and then with 2% aqueous solution of uranyl acetate (UA), pH 4.3 (adjusted by 0.1 *N* NaOH), for 0.5 h, washing briefly in distilled water before and after UA staining.

### Electron Microscopy

Electron microscopy was performed on a JEOL JEM-100 CX-II electron microscope and micrographs were taken at a constant magnification of ×53,000. Grating replicas were used for magnification calibration.

### Measurement of Axial Periodicity

For our calculations, we developed a computer-aided procedure that was based on the periodic variations in intensity along the fibril[15]. Results come from analyses of electron-optical images from untreated and 910-MHz irradiated fibrils.

For statistical analysis, the mean and standard deviation, as well as data from an unpaired *t*-test, were calculated with a laboratory computer according to Snedecor and Cochran[16] and Hays[17]. The significance of the difference between the two samples compared was estimated by using a *p* value following the statistical methods described by Snedecor and Cochran[16].

## RESULTS

Table 1 shows, in the first and third columns of figures, mean values for the axial D periodicity of collagen fibrils from animals that served as controls at two different ages. Small differences among these values are due to the specimen preparation procedure, i.e., the dehydration and the mechanical stresses imposed during sectioning. Irradiated samples showed D spacing significantly ($p = 0$) higher than the control. The only exception is the case of older animals when they were sacrificed at prolonged time after the end of radiation exposure; in this instance the results point to a small increase in D spacing ($p = 0.2$).

**Table 1.**
Periodicity of collagen fibrils sampled from untreated and 910-MHz irradiated tissues. Results are means ± SD (nm)

| | 5 months old | | 16 months old | |
|---|---|---|---|---|
| Time of killing after exposure | control | irradiated | control | irradiated |
| 1 day | 55.3±0.95 | 58.6±1.42 | 54.4±2.53 | 58.9±5.58 |
| 2.5 months | 54.6±2.60 | 58.2±1.40 | 53.4±3.06 | 54.2±3.59 |

### DISCUSSION

Under the present experimental conditions, the results show that 910-MHz electromagnetic radiation affects the axial periodicity of rat arachnoid and dura mater collagen fibrils. As noted earlier, D periodicity in collagen fibrils has its origins in the interactions that occur between the outwardly projecting side chains when two molecules come together in parallel juxtaposition. These intermolecular interactions, particularly those between the larger hydrophobic side chains and those between oppositely charged side chains, are greatest when the adjoining molecules are mutually displaced axially by D or integral multiples of D. As the axial periodicity is affected on exposure, we may conclude that the above interactions might be disturbed by the applied electromagnetic radiation and therefore they are not any more the maximum. Maximum hydrophobic interactions between apolar residues mean maximum hydrogen bonding between excluded water molecules, providing stability to the structure. Other data[18] point to a link between EMF and an evolutionary class of cancer and spontaneous abortion via an increase energy density of chemical bonds that support hydrogen bonds in DNA. Information from another source[19] indicates that 910-MHz electromagnetic radiation has none or a small effect on modifying the charge distribution along the rat arachnoid and dura mater collagen fibrils.

In conclusion, the axial periodicity of collagen fibrils is affected on exposure to 910-MHz electromagnetic radiation as demonstrated by image analysis of electron-optical data. It seems that this influence is restricted to hydrogen bonding between molecules. Providing that this information is coupled with that from other methods, collagen fibril periodicity measurement may serve as an additional marker for the determination of the factors responsible for collagen structural abnormalities brought about by the electromagnetic radiation.

### ACKNOWLEDGMENTS

The authors sincerely thank Dr. Th. Samaras (Radiocommunications Laboratory, Department of Physics, Aristotle University of Thessaloniki, Greece) for valuable help with the FDTD method.
